# 4 Does Addition of Fresh Frozen Plasma to Burn Resuscitation Improve Outcomes in Thermally Injured Patients?

**DOI:** 10.1093/jbcr/iraf019.004

**Published:** 2025-04-01

**Authors:** Joselyn Yang, Todd Huzar, Xu Zhang

**Affiliations:** John S. Dunn Burn Center; McGovern Medical School at UT Health; The University of Texas Health Science Center at Houston

## Abstract

**Introduction:**

Before 2015, isotonic crystalloids and at times, 5% Albumin, were used in fluid resuscitation protocols to treat burn patients at the institution from which this study’s data originates. In 2015, surgeons began resuscitating burns with a combination of crystalloids and FFP. This retrospective study comparatively evaluated whether addition of FFP led to an improvement in patient outcomes when compared to those resuscitated with either crystalloids alone or in combination with 5% albumin. The study focused on these outcomes: total resuscitation volumes, mortality, length of stay, ventilator days, and complications (sepsis, pneumonia, Acute Respiratory Distress Syndrome, renal failure, and Abdominal Compartment Syndrome).

**Methods:**

Using an institution’s EMR and Burn Registry, we comparatively analyzed records from before the implementation of FFP (2009-14) and after (2015-present). The study included patients >18 years old with >20% TBSA burns, admitted to the burn unit. Patients who did not survive >24h, polytrauma patients, chemical or electrical burn patients, or those not admitted to burn unit were excluded. 471 subjects remained and were comparatively matched using a logistic regression model, which included these covariates: age, gender, race, weight, %TBSA, total volume of crystalloid infused, and presence of inhalation injury. Based on this model, we estimated the probabilities for receiving FFP for all subjects and used these as propensity scores. A total of 106 matched pairs were found, with a matched-pairs cohort of 212 subjects. The outcome variables in subjects receiving FFP were compared to the matched subjects who had not received FFP.

**Results:**

The results of the matched pairs cohort (N=212) indicated no significant decrease in resuscitation volumes or difference in mortality, ventilation days, and length of stay between patients with FFP and without FFP. However, patients who received FFP appeared to have lower rates of sepsis (p = 0.040), ARDS (p = 0.012), and renal failure (p = 0.06).

**Conclusions:**

In severely burned patients, this study shows that patients who received FFP as part of their burn resuscitation appeared to have lower rates of developing complications such as sepsis, ARDS, and renal failure.

A limitation of this study is that the effect of FFP was not distinguishable from the temporal effect, which may include innovations in medical technology and other associated treatment methods.

**Applicability of Research to Practice:**

In the past, physicians have relied on isotonic crystalloids for even intravascular and interstitial distribution in burn resuscitation. However, studies show that only 16% of crystalloid volume remains in intravascular space 30 min after infusion. Furthermore, there are concerns of over-resuscitation with crystalloid based formulas, increasing morbidity and mortality. Although many are now using colloid infusions, controversy remains due to lack of statistical evidence supporting clinical outcomes.

**Funding for the Study:**

N/A

